# Complete Coding Region Sequence of a Chikungunya Virus Strain Used for Formulation of CBER/FDA RNA Reference Reagents for Nucleic Acid Testing

**DOI:** 10.1128/genomeA.00587-14

**Published:** 2014-07-31

**Authors:** Germán Añez, Daniel A. Heisey, Maria Rios

**Affiliations:** Laboratory of Emerging Pathogens, Division of Emerging and Transfusion Transmitted Diseases, Office of Blood Research and Review, Center for Biologics Evaluation and Research, U.S. Food and Drug Administration, Bethesda, Maryland, USA

## Abstract

We have sequenced the complete coding region of a chikungunya virus isolate (R91064) used in the formulation of CBER/FDA RNA reference reagents for nucleic acid technology testing.

## GENOME ANNOUNCEMENT

Chikungunya virus (CHIKV) is an alphavirus transmitted by the mosquitoes *Aedes aegypti* and *Aedes albopictus* that has caused explosive epidemics in Africa, Asia, and the Indian and Pacific Ocean islands. There are three recognized CHIKV genotypes: West African, East/Central/South African (ECSA), and Asian (1). By December 2013, CHIKV was detected for the first time in the Americas, more specifically in the island of St. Martin in the Caribbean, where an outbreak has expanded rapidly throughout other areas of the region (2–4). Most CHIKV infections cause an illness characterized by high fever, severe and often incapacitating polyarthralgia, headache, diffuse back pain, myalgia, nausea, vomiting, rash, and conjunctivitis (5). Human CHIKV infections can be asymptomatic, and the virus has been detected in blood samples from donors from the Caribbean, making CHIKV a potential threat for the safety of the blood supply (6). There are no vaccines or specific antiviral treatments for CHIKV. Laboratory diagnosis for CHIKV can be made by serology, viral isolation, or nucleic acid testing (NAT), with NAT being the most sensitive diagnostic method (7). To date, there are no FDA-approved CHIKV diagnostic or blood screening assays. Since the lack of a reference reagent for CHIKV is a barrier for the proper evaluation of NAT assays, we have prepared CBER/FDA reference reagents using a CHIKV isolate imported in 2006 from India to the United States, aiming to assist in the development of NAT assays for diagnostics or blood screening and in regulatory evaluations of assays seeking licensing.

We describe here the complete coding region sequence (CR) of CHIKV strain R91064, for which only a partial genome (partial E2, 6K, and E1 genes) was available (8). Cell culture supernatants from the fourth passage of the virus in Vero cells were used for total RNA extraction using the QIAamp Viral RNA minikit (Qiagen). cDNA was produced with SuperScript III reverse transcriptase (RT) and an oligo(dT)_20_ primer (Invitrogen). Overlapping PCR products were produced with TaKaRa LA polymerase (TaKaRa) and sequenced by the Sanger method. The sequences were assembled and examined using Sequencher version 5.0 (GeneCodes Corp.). The total length of the CR of the virus is 11,237 nucleotides (nt). Partial 5′- and 3′-untranslated region sequences were obtained of 60/76 and 362/513 nucleotides, respectively (numeration based on reference strain S27, accession no. AF369024) (9). BLAST analysis of the sequence obtained revealed that this strain is ca. 99.9% identical (nucleotide and amino acid sequences) to strains that circulated in India during 2006, e.g., IND-06-MH2 and DHS4263-Calif AB (accession no. EF027136 and HM045794, respectively). Phylogenetic analysis conducted in MEGA6 with the maximum-likelihood method and CHIKV CR confirmed that strain R91064 belongs to the ECSA genotype. Due to the abundance of both CHIKV mosquito vectors and susceptible human hosts in the Americas, the establishment of CHIKV in this continent and the occurrence of large outbreaks can be predicted, prompting the urgent need for the correct laboratory identification of these infections.

### Nucleotide sequence accession number.

The complete coding region sequence of the CHIKV strain R91064 has been submitted to the GenBank under the accession no. KJ941050.
